# American Journal of Science and Arts

**Published:** 1858-01

**Authors:** 


### EDITORIAL DEPARTMENT.

*American Journal of Science and Arts.*—The November number of this excellent periodical contains its usual variety of instructive articles. We heartily commend it to the notice of our readers. The following list of its contents will give an idea of the character of the Journal.

Thoughts on species, by T. D. Dana; Researches on the influence of solar light on combustion, by J. Leconte; Influence of sulphur on iron, by Janoyer; Subsidence of United States sea coast, by G. H. Cook; Memoir of Redfield, by D. Olmsted; Zodiacal light, by Rev. G. Jones; New source of electrical excitation, by Mr. Foote; Quantitative assay of chromium by the blow-pipe, by E. W. Hilgard; Fluorine, action of acids on glass, by J. Nicklés; Purification of fluoriferous sulphuric acid, by J. Nicklés; Parthenogenesis; correspondence of J. Nicklés; Scientific intelligence.

